# Three novel *F8* mutations in sporadic haemophilia A cases

**DOI:** 10.1186/2193-1801-1-10

**Published:** 2012-07-30

**Authors:** Rashid Hussain, Noman Bin Abid, Sajjad Hussain, Zeeshan Shaukat, Mudassir Altaf, Sara Altaf, Gulzar Niazi

**Affiliations:** 1 National Institute for Genomics and Advance Biotechnology (NIGAB)/National Agricultural Research Centre (NARC), Park Road, P.O. Box-NIH, Islamabad, Pakistan; 2 Lahore University of Management Sciences, DHA Phase III Hospital Street 29, Lahore, 54792 Pakistan; 3 National Center of Excellence in Molecular Biology, 87-West Canal Bank Road, Thokar Niaz Baig, Lahore, 53700 Pakistan

**Figure 1:**
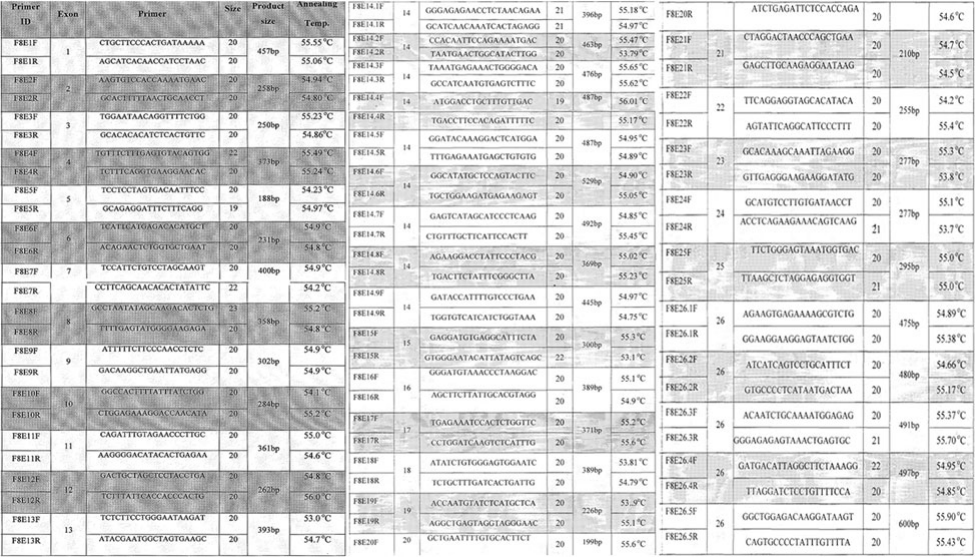
**Primers used in the study.**

Hemophilia A (HA) is an X-linked hereditary disorder characterized by bleeding of variable severity through mild, moderate to severe owing to large range of mutations in the Factor VIII (*F8*) gene (Bowen [2002]). All kind of *F8* mutations, except repeats, have been reported for HA, in total up to 2370 (Human Genome Mutation Database [2005]). A preliminary study was conducted in our lab for identification of mutations in *F8* gene in Pakistani HA patients. Correlation of *F8* mutations with clinical manifestation of HA patients was the main objective of the study. Blood samples were collected from 62 HA patients from all over the Pakistan and clinical history of all HA patients was recorded (only patients frequently visiting medical centers for the replacement of Factor VIII were selected for the study). Genomic DNA was extracted from whole blood by standard organic procedure. Specific primers (Figure 1) were designed using “Primer3” (http://biotools.umassmed.edu/bioapps/primer3_www.cgi) to amplify the coding region of *F8* gene; amplified products were sequenced by ABI 310 and ABI 3100 sequencer (Applied Biosystems, Carlsbad, CA, USA). The sequencing results were visualized using “Chromas 2.33” software (Applied Biosystems) and mutations were detected using “BLAST” software available on the NCBI website (http://http:balst.ncbi.nlm.nih.gov/Blast.cgi). Three novel mutations (1 deletion; 2 point mutations) were detected in four sporadic HA patients, all from different ethnic backgrounds (Table 1**)**. The deletion of T in exon 7 within the A1 domain represents a frame-shift change disrupting the protein structure and function, which result in severe manifestation of the disease. A missense point mutation in the A3 domain occurs in codon 1907 at nucleotide number 5720, replacing Serine with Isoleucine, and confers a moderate type of severity. It should be noted that Serine is a polar and acidic amino acid while Isoleucine is a nonpolar and basic amino acid. A nonsense point-mutation was found in two unrelated patients in the C3 domain (exon 26) and was correlated with moderate clinical findings. Beside these mutations, 27 common SNPs were also detected in *F8* gene for the studied patients (Table 2). The allelic data and accession numbers of these SNPs were collected from Ensembl Genome Browser (Ensembl [2000]). The results of the study will form the basis not only for an enlarged study but also for diagnosis and genetic counseling of classical hemophilia in Pakistan.

**Table 1:** **Novel mutations in*F8*gene**

Age/Sex	Severity	Exon	Nucleotide change	Amino acid change	Codon/Codon no.	Nucleotide genome ref./cDNA ref.	Affected Domain
4 yr /male	Severe	7	Deletion of T	Frame-shift	CTC → C-C/ 318	159197688/953	A1
35 yr / male	Moderate	17	G → T	Ser → Ile	AGC → ATC/ 1907	154132724/5720	A3
15 & 19 yr /male	Moderate	26	C → A	Tyr → Termination	TAC → TAA/ 2324	154065994/6972	C2

*yr (years)*.

**Table 2:** **Common SNPs in*F8*gene (exonic region)**

Sr. #	Patient	Exon	SNP ambiguity	SNP	Codon	Codon#	Comments	Accession number
1	All 62 Samples	2	W: A/T	A/A	GAT	75	European = T/T	rs1800288
2	All 62 Samples	7	K: G/T	G/G	TGG	274	European = C/C; Spanish Caucasians = C(0.995)/A(0.005); African American, Chinese, Southeast Asia, Mexican Indian = C/A	rs34371500
3	All 62 Samples	8	R: G/A	G/G	CGC	391	Ancestral: **G**	rs137852364
4	All 62 Samples	8	Y: T/C	T/T	TCA	392	European = C/C	rs28933669
5	All 62 Samples	8	Y: C/T	C/C	TCA	392	?	rs28933668
6	All 62 Samples	8	K: T/G	T/T	ATT	405	European = A/A	rs28933670
7	All 62 Samples	8	R: A/G	A/A	GAG	409	?	rs28933671
8	All 62 Samples	9	K: G/T	T/T	TTG	431	Ancestral: G	rs28933672
9	All 62 Samples	9	R: A/G	A/A	AAA	444	Ancestral: **G**	rs28937272
10	All 62 Samples	9	W: T/A	T/T	TAC	450	Ancestral: **A**	rs111033616
11	All 62 Samples	10	R: G/A	G/G	CGT	503	Ancestral: **A**	rs35383156
12	All 62 Samples	12	Y: T/C	T/T	CTT	622	Ancestral: **T**	rs1800290
13	All 62 samples	15	R: G/A	G/G	CAG	1764	Ancestral: **A**	rs5986891
14	All 62 samples	16	R: G/A	G/G	ATG	1842	European = G/G	rs28943674
15	All 62 samples	16	Y: C/T	C/C	CCC	1844	European = C/C	rs28933675
16	All 62 samples	16	M: A/C	A/A	ACT	1845	?	rs28933676
17	All 62 samples	16	Y: C/T	C/C	GCC	1853	European = C/C	rs28933677
18	All 62 samples	17	D: G/A/T	G/G	GAT	1865	Not Available	CI076951
19	All 62 samples	17	R: A/G	A/A	CAC	1867	Ancestral: **G**	rs28933679
20	All 62 samples	17	S: C/G	C/C	CCC	1873	European = G/G	rs28933680
21	All 62 samples	17	R: G/A	G/G	GAG	1904	European = C/C	rs28933681
22	All 62 samples	17	S: G/C	G/G	TGC	1922	European = G/G	rs4384155
23	All 62 samples	17	S: C/G	C/C	TGC	1922	European = C/C	rs4520342
24	All 62 samples	18	R: A/G	A/A	AAT	1940	?	CM083806
25	All 62 samples	18	D: G/A/T	G/G	CGA	1960	?	rs28937294
26	All 62 samples	18	R: G/A	G/G	GGC	1967	?	rs111033615
27	All 62 samples	24	Y: C/T	C/C	TAC	2214	Ancestral: **C**	rs1800296
